# The Effect of Deltamethrin-treated Net Fencing around Cattle Enclosures on Outdoor-biting Mosquitoes in Kumasi, Ghana

**DOI:** 10.1371/journal.pone.0045794

**Published:** 2012-09-19

**Authors:** Marta Ferreira Maia, Ayimbire Abonuusum, Lena Maria Lorenz, Peter-Henning Clausen, Burkhard Bauer, Rolf Garms, Thomas Kruppa

**Affiliations:** 1 London School of Hygiene and Tropical Medicine, Department of Disease Control, London, United Kingdom; 2 Ifakara Health Institute, Bagamoyo, Pwani Region, United Republic of Tanzania; 3 Kumasi Centre for Collaborative Research in Tropical Medicine, Kumasi, Ghana; 4 Bernhard-Nocht Institute for Tropical Medicine, Hamburg, Germany; 5 Free University of Berlin, Faculty of Veterinary Medicine Institute for Parasitology and Tropical Veterinary Medicine, Berlin, Germany; Johns Hopkins University, United States of America

## Abstract

Classic vector control strategies target mosquitoes indoors as the main transmitters of malaria are indoor-biting and –resting mosquitoes. However, the intensive use of insecticide-treated bed-nets (ITNs) and indoor residual spraying have put selective pressure on mosquitoes to adapt in order to obtain human blood meals. Thus, early-evening and outdoor vector activity is becoming an increasing concern. This study assessed the effect of a deltamethrin-treated net (100 mg/m^2^) attached to a one-meter high fence around outdoor cattle enclosures on the number of mosquitoes landing on humans. Mosquitoes were collected from four cattle enclosures: Pen A – with cattle and no net; B – with cattle and protected by an untreated net; C – with cattle and protected by a deltamethrin-treated net; D – no cattle and no net. A total of 3217 culicines and 1017 anophelines were collected, of which 388 were *Anopheles gambiae* and 629 *An. ziemanni*. In the absence of cattle nearly 3 times more *An. gambiae* (p<0.0001) landed on humans. The deltamethrin-treated net significantly reduced (nearly three-fold, p<0.0001) culicine landings inside enclosures. The sporozoite rate of the zoophilic *An. ziemanni*, known to be a secondary malaria vector, was as high as that of the most competent vector *An. gambiae*; raising the potential of zoophilic species as secondary malaria vectors. After deployment of the ITNs a deltamethrin persistence of 9 months was observed despite exposure to African weather conditions. The outdoor use of ITNs resulted in a significant reduction of host-seeking culicines inside enclosures. Further studies investigating the effectiveness and spatial repellence of ITNs around other outdoor sites, such as bars and cooking areas, as well as their direct effect on vector-borne disease transmission are needed to evaluate its potential as an appropriate outdoor vector control tool for rural Africa.

## Introduction

Within the last decade, great advances have been made in the fight against vector borne diseases with malaria decreasing considerably in sub-Saharan Africa [1], [2], [3], [4]. However, despite well-planned vector control programs, diagnostics and artemisinin-combination therapies (ACTs), malaria transmission continues to persist in some settings. A large-scale integrated malaria control intervention was implemented on Bioko Island, a small, contained island situated off the coast of Equatorial Guinea, to eliminate the disease [5]. However, contrary to the classical behaviour pattern of *Anopheles gambiae* sensu stricto (s.s.), a large proportion of malaria vectors were found biting outdoors, and malaria elimination failed [6]. This has also been observed in other settings suggesting that the application of vector control tools that strictly and aggressively target mosquitoes biting and resting indoors may increasingly shift malaria transmission loci from in-to outdoors [7], [8]. This may occur due to genotype bottlenecking of individuals within a species under selection from insecticidal pressure from Insecticide-Treated Nets (ITNs) and Indoor Residual Spraying (IRS), favouring those vectors that adapt to outdoor or early biting, or through species replacement. Recently, a cryptic subgroup of wholly exophilic *An. gambiae* was found in West Africa, reminding the scientific community on how little is known, or how much is assumed, on the diversity and behaviour of this species complex [9]. In addition, several regions have reported an increase in malaria transmission by secondary malaria vectors [10], [11]. Most secondary malaria vectors are known to feed outdoors and preferably on animals, this way eluding indoor vector control interventions. Their zoophagy allows them to find refuge in cattle and maintain vectorial fitness despite interventions. There is only limited knowledge of the dynamics of vectorial systems and their contribution to malaria transmission after the implementation of large-scale interventions targeting indoor feeding vectors. Local and up-to-date information on vector systems is essential to understand malaria epidemiology and design appropriate vector control programs.

In order to tackle malaria elimination and the elimination of other neglected tropical diseases such as filariasis and dengue, new innovative outdoor vector control tools must be developed. One of the only methods of protection against outdoor-biting mosquitoes are topical repellents [12]. These require regular compliance by the user and offer only individual protection. In contrast, spatial or area repellents create a protective area by volatilizing repellent or low-dose insecticides into the air, thus enabling the protection of several individuals within an area. Mosquito coils are the classical example of a spatial repellent; pyrethroids are dispersed into the air through slow volatilization when the coil is lit. Similarly to topical repellents, this intervention requires nightly compliance as well as regular purchasing. Alternatively, pyrethroid-treated nets, besides killing mosquitoes, also repel them [13], [14], [15]. One of the most successful vector control interventions in history was IRS using DDT, which also works as a spatial repellent by deterring mosquitoes from sprayed households. The use of spatial repellency combined with insecticidal toxicity could reduce insect pressure outdoors if a pyrethroid-treated net is placed strategically at locations where people gather in the evening, for example a public venue or an outdoor domestic area.

In the present study, cattle enclosures were used to simulate a highly attractive outdoor area to measure whether the outdoor application of a pyrethroid-treated net 1) reduces mosquito densities within the enclosures, and 2) reduces mosquitoes in its vicinity through spatial repellence. In addition, cattle can be used to divert mosquitoes from humans to cows (zooprophylaxis). This has been applied with variable success in the past, with some cases resulting in increased mosquito densities (zoopotentiation) rather than zooprophylaxis, as the availability of blood meals increases the probability of mosquito survival and fecundity [16], [17], [18], [19]. However, the combination of zooprophylaxis and chemical control may present a valuable strategy for controlling outdoor biting mosquitoes [20], [21]. Studies have shown that by regularly treating cattle with insecticidal pour-on, mosquito densities and disease incidence can be reduced [22]. Additionally, usage of pour-on solutions increases the cost-effectiveness of animal production for farmers through control of ticks and other harmful insects [23], [24], [25]. The use of ITNs around cattle stables reduces nuisance and biting flies attacking enclosed animals by 80% compared to controls [26]. This new method of deploying ITNs may provide a low-cost alternative to insecticide-treated cattle (ITC) and become a further feasible vector-control tool in rural sub-Saharan Africa.

## Methods

**Figure 1:**
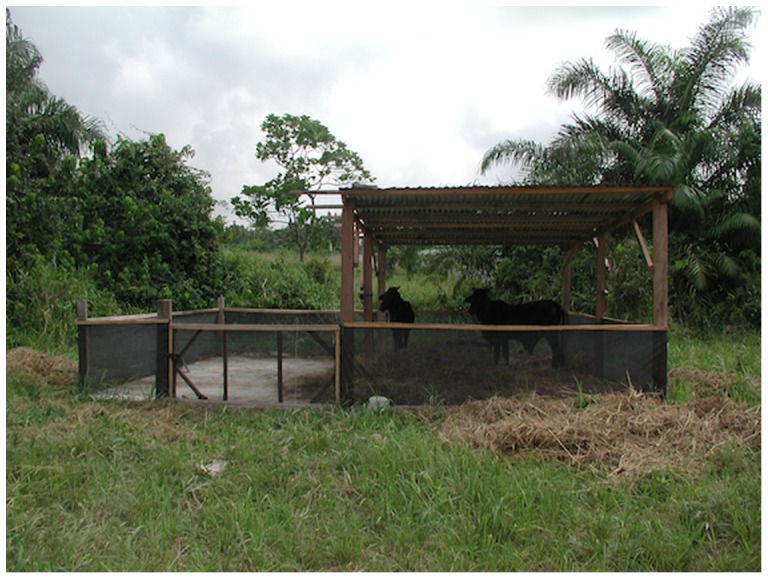
Experimental animal enclosure at Boadi Cattle Research Farm, KNUST.

The study took place in Kumasi, Ghana at the Boadi Cattle Research Farm of the Kwame Nkrumah University for Science and Technology (KNUST). The study was conducted for six weeks during the months of October and November 2005. This period corresponded to the end of the rainy season; mosquito breeding sites were identified in the area prior to study initiation. Four similar sites located 500 m from each other were chosen, within which four identical cattle pens were built (Figure 1). The pen dimensions were 6×7 meters; the floor consisted of concrete and half of the pen was roofed with corrugated iron sheets. All pens were fenced with 1 m-high chicken wire. Pens were randomly assigned a treatment:

Pen A – No netting protection and occupied by two zebu No netting and no animals.
Pen B – Protected by an untreated net attached to the chicken wire fence (1 meter height) and occupied by two zebu.
Pen C – Protected by the same but deltamethrin-treated net – 100 mg/m^2^ – attached to the chicken wire fence (1 meter height) and occupied by two zebu.
Pen D –No netting protection and occupied by two zebu.

In all pens except pen D, two zebu bulls of comparable size and colour were introduced and kept in a zero-grazing system. These were weekly rotated to prevent biases due to differences in individual attraction. The netting material consisted of a black color, polyester fiber with a mesh width of 2×2 mm (Vestergaard-Frandsen Lausanne, Switzerland). The manufacturer incorporated an UV protection factor in the treated net to prevent the decay of deltamethrin by exposure to sunlight.

During 6 weeks, human landing catches (HLCs) were performed once a week within and 20 m away from each pen. Sixteen volunteers were divided into two shifts and performed HLCs from 18:00 to 06:00. Landing rates were recorded for each hour. An on-site weather station measured precipitation and minimum and maximum temperature during each collection night. Caught mosquitoes were taken to the Kumasi Centre for Collaborative Research (KCCR) where they were morphologically identified to culicines and anophelines. The latter were identified to species using morphological keys [27]. All the collected *Anopheles* females were examined for the presence of circumsporozoite *Plasmodium falciparum* antigen using head and thorax with enzyme-linked immunosorbent assay (ELISA)[28]. Sporozoite rates (SR) were calculated per location by dividing the number of infected mosquitoes by the total of mosquitoes caught each month. Daily entomological inoculation rates (EIR) were calculated for each study site by multiplying the sporozoite rate by the average human biting rate per night.

Samples from the deltamethrin-treated net in pen C were regularly taken for a period of nine months after project start and submitted to bioassays using lab-reared *Aedes aegypti.* Persistence of insecticidal activity in the treated net exposed to outdoor conditions was tested. Bioassays were conducted by lining the inside of a small cylindrical container with experimental net (5 cm diameter×10 cm height). The control container was lined with non-impregnated net. All net samples were tested twice; 30 mosquitoes were inserted into the container through a small hole and submitted to 10 seconds contact with the net. The mosquitoes were then released into a large cage and monitored for knockdown and mortality after 5 minutes, 10 minutes, 15 minutes, 6 hours and 24 hours.

Data analysis was performed using package ‘lme4’ [29] in R [30]. The total numbers of female mosquitoes caught by HLC were analysed separately for the three groups of mosquitoes (culicines, *An. gambiae* and *An. ziemanni*) with Poisson-lognormal mixed-effects models to account for over-dispersion in the count data. Model estimated means (MEM) were calculated using this model and used for comparison between pens. Treatment was set as the fixed effect and experimental day was set as the random effect. The reference pen was considered to be the unprotected pen with cattle (pen A). Comparison between pens A (cattle) and D (no cattle) showed whether zooprophylaxis was occurring. Comparisons between pens A (no net), B (untreated net), and C (treated net), respectively showed the effect of a deltamethrin-treated net on the density of host-seeking mosquitoes. Separate analyses were performed for mosquitoes caught 1) indoors and 2) 20 m apart from the enclosures in order to be able to differentiate spatial repellence effects.

Ethical approval was obtained from Kumasi Centre of Collaborative Research Institutional Review Board (KCCR–IRB), certificate number: KCCR/IRB/063/11. Participants were enrolled on verbal informed consent as approved by the KCCR-IRB, this was chosen given the fact that most volunteers did not speak English and could not read and write. Following the IRB approved protocol a meeting was held with all participants in their local language explaining the project outline and participatory risk. The meeting was documented with the signature of all participants and of two witnesses. Diagnosis and treatment were offered during the entire period of the project and one month after conclusion, though none of the volunteers became sick during or at least four weeks after the catching period.

## Results

Human landing catches collected 3217 culicines and 1017 anophelines of which 388 were *An. gambiae* and 629 *An. ziemanni*. Other studies conducted in the area attest that the only *An. gambiae* complex species present in Kumasi is *An. gambiae* s.s. [31], [32]. In addition, PCR analysis of mosquitoes collected from the same study site a few months later confirmed the sole presence of *An. gambiae* s.s. (Abonuusum, unpublished). *Anopheles gambiae* showed a biting peak between 02:00 and 04:00 compared to *An. ziemanni*, which fed mostly between 00:00 and 02:00. The cumulative proportion of human landings between 18:00 and 22:00 was 9.3% and 18.3% for *An. gambiae* and *An. ziemanni* respectively. The presence of cattle reduced the number of human-host-seeking *An. gambiae* inside the enclosures by 66% (p<0.0001) (Table 1). The same was not observed for *An. ziemanni* or culicines. Cattle presence did not influence the number of mosquitoes caught 20 m from the enclosures (Table 2).

**Table 1:** Total number of mosquitoes, model estimated means (MEM) and 95% confidence intervals (95%CI) of HLC collections performed inside all experimental pens as well as sporozoite rates (SR) and entomological inoculation rates (EIR).

Species and location	N	MEM^1^	95% CI	p value	SR%	EIR^2^
*Anopheles gambiae*						
Pen A – Cattle, no net 3	42	5.97	(3.24–10.97)	–	0.00	0.0
Pen B –Cattle, untreated net	51	6.84	(3.93–11.90)	0.629	1.96	0.1
Pen C – Cattle, treated net	28	3.76	(2.04–6.92)	0.138	3.57	0.1
Pen D – No Cattle, no net	121	17.54	(10.56–29.10)	<0.0001 ***	2.48	0.4
*Anopheles ziemanni*						
Pen A – Cattle, no net 3	28	3.65	(1.89–7.04)	–	0.00	0.0
Pen B –Cattle, untreated net	57	7.85	(4.00–15.39)	0.0257 *	0.00	0.0
Pen C – Cattle, treated net	32	4.39	(2.15–8.97)	0.614	3.13	0.1
Pen D – No Cattle, no net	18	2.56	(1.18–5.56)	0.370	0.00	0.0
Culicines						
Pen A – Cattle, no net 3	348	50.43	(35.64–71.36)	–	–	–
Pen B – Cattle, untreated net	514	80.73	(58.88–110.68)	0.0035 **	–	–
Pen C – Cattle, treated net	128	19.84	(13.96–28.19)	<0.0001 ***	–	–
Pen D – No Cattle, no net	379	60.66	(44.07–83.49)	0.257	–	–

1 Model estimated mean.

2 Daily EIR – number of infected bites per person per night.

3 Reference Pen is A – no net and occupied by two zebu bulls.

**Table 2:** Total number of mosquitoes, model estimated means (MEM) and 95% confidence intervals (95% CI) of HLC collections performed 20 m apart from all experimental pens as well as sporozoite rates (SR) and entomological inoculation rates (EIR).

Species and location	N	MEM^1^	95%CI	p value	SR%	EIR^2^
*Anopheles gambiae*						
Pen A – Cattle, no net 3	45	5.83	(2.98–11.40)	–	2.20	0.1
Pen B –Cattle, untreated net	37	4.85	(2.53–9.30)	0.579	2.70	0.1
Pen C – Cattle, treated net	33	4.30	(2.22–8.33)	0.366	0.00	0.00
Pen D – No Cattle, no net	31	4.15	(2.13–8.09)	0.319	3.23	0.1
*Anopheles ziemanni*						
Pen A – Cattle, no net 3	117	14.70	(8.15–26.51)	–	1.71	0.3
Pen B –Cattle, untreated net	125	17.00	(8.54–33.84)	0.680	1.60	0.3
Pen C – Cattle, treated net	77	10.01	(4.93–20.32)	0.287	1.30	0.1
Pen D – No Cattle, no net	175	25.08	(12.73–49.42)	0.123	2.29	0.6
Culicines						
Pen A – Cattle, no net 3	405	63.31	(47.59–84.24)	–	–	–
Pen B –Cattle, untreated net	656	102.16	(78.10–133.62)	0.0005 ***	–	–
Pen C – Cattle, treated net	321	52.09	(39.40–68.86)	0.171	–	–
Pen D – No Cattle, no net	520	82.24	(62.70–107.87)	0.059	–	–

1 Model estimated mean.

2 Daily EIR – number of infected bites per person per night.

3 Reference Pen is A – no net and occupied by two zebu bulls.

The HLCs performed inside the pen surrounded by the untreated net collected the highest number of *An. ziemanni* (N = 57; MEM = 7.85; p = 0.0257) and culicines (N = 514; MEM = 80.73; p = 0.0035; Table 1) compared to the reference pen that contained animals and was not protected by any type of netting. The model estimated mean of culicine human landings was 38% higher inside the pen (p = 0.0035) and 38% higher outside the pen (p = 0.0005; Table 1 and 2) surrounded by an untreated net than when the pen had no netting protection. On the other hand, the insecticide-treated net considerably reduced the density of host seeking culicines by 61% (N = 128; MEM = 19.84; p<0.0001) compared to the pen with cattle and no protection (Table 1). There was no effect of ITN use on mosquitoes collected 20 m from the pens, regardless of mosquito species (Table 2).

Sporozoite incrimination revealed infection in both *An. gambiae* and *An. ziemanni* with sporozoite rates (SR) ranging from 0 to 3.57% (mean = 2.02%) in *An. gambiae* and 0 to 3.13% in *An. ziemanni* (mean = 1.25%) (Table 1 and 2). The daily EIRs were calculated for both species. *Anopheles ziemanni* scored the highest EIR with 0.6 infective bites per person per night compared to 0.4 for *An. gambiae* (Table 1 and 2).

Bioassays using the ITN deployed for the study were conducted using lab-reared *Aedes aegypti*. More than 80% of the mosquitoes exposed to the treated nets were paralyzed after 15 minutes, with the exception of the net sampled 9 months later, where only 40% were paralyzed within 15 minutes (Table 3). However, even after 9 months of exposure, more than 80% of the mosquitoes were paralyzed after 6 hours. The percentage of recovering mosquitoes after 24 hours remained below 5% up to 9 months, after which it increased to 20%.

**Table 3:** Percentage of active *Aedes aegypti* following 10 seconds exposure to the treated net samples collected from the field after 5 minutes, 10 minutes, 15 minutes, 6 hours and 24 hours.

Time after exposure	Control	2 months	5 months	7 months	8 months	9 months
5 min	98	97	94	97	94	99
10 min	98	61	49	17	47	83
15 min	98	6	0	2	19	59
6 h	97	0	0	0	2	16
24 h	93	0	4	0	2	16

## Discussion

In the present study, the presence of cattle reduced the *An. gambiae* human biting rate inside the pens by 66% (p<0.0001), indicating that either mosquitoes were diverted from humans to cattle or *An. gambiae* were repelled by the cattle odours [33]. On the other hand, the animal presence attracted higher densities of zoophilic mosquitoes like *An. ziemanni* and diverse culicines, which then had the choice of a human or animal blood meal. The number of host-seeking culicines inside the pen protected by the untreated net was significantly higher than in the unprotected pen (p = 0.0035), the same was observed for *Anopheles ziemanni* (p = 0.0257). The untreated net might have acted as a physical barrier hindering mosquitoes that entered the pen from leaving. The ITN surrounding the cattle enclosure provided an added protection from nuisance mosquitoes, resulting in 61% fewer host seeking culicine mosquitoes (p<0.001) and 37% fewer *An. gambiae* (p = 0.138) inside the enclosures. Results for anophelines were not significant, probably because the number of *Anopheles* mosquitoes being caught was low and the study was therefore underpowered for malaria vectors. However, our results indicate that the introduction of ITN fences around animal enclosures could result in fewer host-seeking mosquitoes inside the enclosure. The insecticidal effect of deltamethrin remained effective for the study period of nine months following deployment despite the net material being exposed to weathering and sunlight. Twenty-four hours post-exposure to the treated net that had been in the field for nine months yielded 84% knockdown of tested *Aedes aegypti* mosquitoes. Further experiments should be devised to test the longevity of the insecticidal activity of this netting material against lab-reared malaria vectors. Though it is expected that results will be encouraging since *Aedes aegypti* are a very robust mosquito specimen, usually needing a stronger stimulus for knockdown than Anophelines.

Outdoor transmission of vector borne diseases is an increasingly important problem that needs to be addressed in order to reach disease elimination [6], [7]. Traditional methods such as IRS and ITNs target mainly indoor-biting vectors and neglect outdoor-biting mosquitoes, for which insufficient tools are available. In Pakistan, the usefulness of zooprophylaxis as a malaria vector control tool was studied in a community-randomised clinical trial. Regular treatment of cattle with deltamethrin pour-on resulted in over 50% reduction of clinical malaria episodes [22]. The major disadvantages of ITC (insecticide-treated cattle) are the necessary regular re-treatments, reliance on compliance and the constant community-wise investment. Additionally, mistreatments, such as substituting pour-on solutions with old motor oil and household disinfectants, could jeopardize successful vector control [25]. In Kenya, ITNs were used to protect enclosed cattle from tsetse flies, which successfully reduced animal morbidity. As an additional benefit, villagers also reported fewer mosquitoes in their homes [34]. More recently tsetse flies were successfully controlled in the Eastern Region of Ghana [35] The use of ITNs surrounding cattle enclosures would provide a less costly alternative to ITC and, since the insecticidal activity lasted over nine months, a long-lasting tool for vector control. However, its application would require that cattle were either kept enclosed in zero-grazing units or summoned indoors in the evenings, potentially limiting the application of insecticide-treated net fencing to locations where husbandry practices permit.

At the community level, a proportion of the mosquitoes seeking blood meals could be diverted from the cattle enclosures to humans due to a repellent effect of deltamethrin-treated nets. So far only one study has attempted to measure the diversion of mosquitoes from ITCs to humans [36]. However the methodology employed did not investigate the possibility of the treated-cattle acting as a source of spatial repellence since the distance between HLC and cattle was only two meters [33]. Spatial repellence of mosquito coils and ITCs works by exuding a chemical barrier that protects not only the cows from mosquitoes, but also everything else within a certain radius. Thus, it is impossible to evaluate mosquito diversion if measurements are made only at short distances from the repellent source. There is an urgent need to evaluate whether spatial repellents result in mosquito diversion to non-users. In this study, the number of mosquitoes caught 20 m away from the cattle enclosure did not differ between pens without a net or with ITNs. Therefore, no spatial repellence was measured in Kumasi. The 20 m distance chosen in this study may have been too far away from the cattle enclosures for this ITN brand. The protective radius of deltamethrin-treated nets (100 mg/m^2^) is as yet unknown, so a range of distances from five to 20 meters for mosquito catches should be tested in further experiments. In addition, here, treatments were fixed to each location and not rotated. Future studies must consider this source of spatial variation and both replicate and rotate treatments between sites.

Many malaria elimination scenarios around Africa are confronted with the increase in secondary malaria vectors or species replacement due to selective pressure from the existing malaria control tools. In some areas, *An. arabiensis* have replaced the classical main vectors, *An. gambiae* s.s. and *An. funestus*, and are now considered the major malaria vectors [7], [37]. *Anopheles arabiensis* presents highly variable feeding behaviour dependant on climate and location: it has been shown to feed on blood meals both from humans and animals, with early evening and late-evening habits [7], [38], [39], [40]; these characteristics make it more difficult to control through indoor-targeted interventions. In southern Zambia, despite high bed-net coverage *An. arabiensis* remained highly anthropophilic with less than 6% of analysed blood-meals being taken from animal origin [41], proving that the behaviour of the local *An. arabiensis* precluded malaria control through ITN usage. In the present study, *An. ziemanni*, a zoophilic mosquito [42], was caught in very high numbers on human landings. Host seeking *An. ziemanni* were mostly caught outside the animal pens, which is consistent with the outdoor-biting behaviour of this species [42]. However, *An. ziemanni* has not been known to readily feed on humans as was observed in this study. A considerable number of *An. ziemanni* were collected outside the enclosure that did not contain animals, indicating that *An. ziemanni* were probably attracted to the humans despite the absence of cattle odour cues. High numbers of *An. ziemanni* were also collected on humans sitting outside pens containing animals; in this case the mosquito had been given the choice between an animal or human blood meal and chose to bite the human host. In addition, sporozoite incrimination revealed surprisingly high sporozoite rates and EIR for this species (Table 2). The cumulative proportion of *An. ziemanni* bites from dusk until 22∶00 was 18.3%; during this period people will not yet have retired to the protection of their bed net and might be exposed to the activity of outdoor-biting malaria vectors. In areas where people spend most of their evening time outdoors and cattle are present, *An. ziemanni* might be responsible for a much higher proportion of malaria transmission than previously expected through zoopotentiation. As such, these findings provide further evidence towards the notion that changes are occurring in malaria transmission in sub-Saharan Africa and call attention to new potential secondary vectors.

Ideally, ITN fences should be evaluated inside a semi-field system, where safe and controlled conditions allow a high-throughput method to measure spatial repellence, feeding inhibition, protective distance and delayed mortality. Other compounds or compound combinations aside from deltamethrin should also be evaluated, such as transfluthrin, metofluthrin or actellic. This experiment would grant a holistic perception on the impact of ITN fencing on host-seeking malaria vectors and other mosquito species. The results arising from such an evaluation would form the foundation to further studies investigating the efficacy of ITNs around outdoor sites, such as bars and cooking areas. Studies should be performed for longer periods of time to achieve sufficient power as well over different seasons of the year. If successful this intervention could present an innovative and appropriate tool against outdoor-biting and outdoor-resting disease vectors in rural Africa.
